# Patients’ experience of accessing support for tics from primary care in the UK: an online mixed-methods survey

**DOI:** 10.1186/s12913-023-09753-5

**Published:** 2023-07-24

**Authors:** Christina Marino, Kareem Khan, Madeleine J Groom, Sophie S Hall, Seonaid Anderson, Emma Mcnally, Tara Murphy, Charlotte L Hall

**Affiliations:** 1 School of Medicine, Leicester Medical School, College of Life Sciences, University of Leicester, Leicester, UK; 2 Mental Health & Clinical Neurosciences, NIHR Nottingham Biomedical Research Centre, School of Medicine, NIHR MindTech Medtech Co-operative, University of Nottingham, Nottingham, UK; 3 Nottingham Clinical Trials Unit, School of Medicine, University of Nottingham, Nottingham, UK; 4 Neurodiverse.org, Brussels, Belgium; 5 Tourettes Action, Farnborough, UK; 6 Tic Disorder Service, PAMHS, Great Ormond Street Hospital, London, UK

**Keywords:** Tics, Tourette syndrome, GP, Adults, Young people, Primary care, UK, Online survey

## Abstract

**Background:**

Tics are common in children and young people and may persist into adulthood. Tics can cause challenges with social, occupational, physical, and academic functioning. The current study explores the perceptions of adults with tics and parents/carers of young people with tics regarding their experience of accessing support from professionals in primary care in the UK.

**Methods:**

Two online cross-sectional surveys were completed by 33 adults with tics and 94 parents/carers of children with tics. Participants were recruited across three online tic support groups. Tic specialist psychologists, academic researchers, and people with lived experience of tics provided feedback on the surveys before they were made available online. Mixed-method analyses were conducted on the surveys. Qualitative data from the free-text responses were analysed using thematic analysis and triangulated with quantitative findings where appropriate.

**Results:**

While some participants felt supported by general practitioners (GPs), many felt dismissed. The impact of tics was not always explored, nor information on tics provided, during the consultation. Although 78.7% of participants were referred to secondary care for their tics, some struggled to get the referral. Within secondary care, most adult respondents were assessed by neurologists whilst young people were typically assessed by paediatricians or psychiatrists. Most of these secondary care clinicians did not specialise in tic disorders, with only 27.9% of participants being assessed by tic specialists. Mode waitlist time was 3–6 months for young people and longer for adult respondents. Some participants were referred to multiple secondary care services, spanning neurology, paediatrics, and psychiatry, with each stating that they do not provide support for tics. 21% of participants mentioned being discharged from secondary care with no ongoing support. Almost one-third of respondents accessed support within private healthcare.

**Conclusions:**

Generally, more negative than positive experiences were reported. Possible contributing factors included a lack of clear tic referral pathways, long waitlists, a lack of information about tics provided in primary care appointments and a lack of support offered following diagnosis by secondary care services, together with poor access to tic specialist clinicians. This study highlights areas where improvements to UK services for tics can be made.

**Supplementary Information:**

The online version contains supplementary material available at 10.1186/s12913-023-09753-5.

## Background

Tics are brief, non-rhythmic movements or sounds which are either involuntary or semi-voluntary [1], meaning they can be difficult to control. Up to 20% of the population experience tics during childhood [2], with a higher prevalence seen in boys than girls in a ratio of approximately 4:1 respectively [2–4]. Tics that are present for less than one year may be given a diagnosis of provisional tic disorder, whilst those which remain longer fit diagnoses of chronic tic disorder (when either motor or vocal tics are present) or Tourette syndrome (TS) (when both motor and vocal tics are present), which have a combined prevalence of 2.5% [4]. Tics often reach peak severity in early adolescence [1, 5]. Previous expert consensus stated that tics persist into adulthood in a minority of cases [6–8]. However, recent studies have found that complete remission of tics occurs in only up to one-third of individuals within a decade of first tic onset [9, 10] and a minority experience worsening of their tics [11]. Psychiatric co-morbidity is common, with some studies showing that up to 50% of people with TS have attention/deficit hyperactivity disorder (ADHD) [12, 13] or obsessive-compulsive disorder (OCD) [14, 15], and approximately one-third experience anxiety [16] or mood disorders [13, 17]. Having long-standing tics is associated with a fourfold increased risk of death by suicide [18]. The impact of tics can be variable, affecting academic [19], social [20–22], occupational [19], and physical functioning due to pain [23, 24]. Without adequate support, these factors can contribute to lower quality of life [1].

In the UK, individuals seeking support for their tics within the National Health Service (NHS) must first attend an appointment with a General Practitioner (GP) working in primary care. If the GP suspects the presence of tics and recognises that the patient’s need falls outside of their scope of practice, a referral can be made to NHS secondary care services. This high level of need is not atypical, as a full assessment and management of tics and co-occurring conditions frequently requires input from multidisciplinary team members [25], meaning that tics cannot always be managed by GPs alone. Referrals may be made to Child and Adolescent Mental Health Services (CAMHS), general (or community) paediatricians, paediatric neurologists, adult psychiatrists, or adult neurologists. These secondary care specialists may or may not have expertise in tics. According to international guidelines [26–28], the three main management options for tics include psychoeducation, behavioural therapy, and pharmacotherapy. However, in the UK, only 1 in 5 children and young people can access behavioural therapy for tics due to a lack of trained therapists [29].

Relatively little literature has explored patients’ experience of accessing healthcare services for support for their tics. Two studies [29, 30] have highlighted a perception among patients that healthcare professionals have insufficient knowledge about TS. However, as the term ‘healthcare professional’ (HCP) encompasses a variety of staff members, it is unclear from the research who the HCPs were. In a survey of 295 parents living in the UK, 10% commented that their GP had a limited understanding of tics, and one-third reported difficulty in getting a referral to secondary care for their child’s tics [29]. In contrast, 9.5% of respondents stated that their GP immediately referred their child to secondary care. This could indicate variation in GPs’ ability to identify tics or local variability in the availability of secondary care services for tics which GPs can refer on to. Data collected from 28 UK secondary care professionals found that most patients were offered an appointment 3–6 months after the initial GP referral [31]. This finding of long wait times to access UK secondary care services for tics was also reported by Cuenca et al. [29], who found that 16.3% of surveyed parents described getting a diagnosis for tics for their child as a lengthy and difficult process. However, what this difficult process entailed is not described.

Healthcare services in the UK typically follow recommendations from the National Institute for Health and Care Excellence (NICE) to set standards of care [32]. Whilst there are UK guidelines from NICE for the assessment, referral, diagnosis, and management of other neurodevelopmental conditions such as ADHD [33] and autism [34–36], similar comprehensive guidance does not exist for tics. However, the lack of NICE guidance to provide recommendations for the initial assessment of tics within primary care and the recommended scope of practice for secondary care services for tics means there is a potential for differential expertise, access, and availability of services for patients with tics across the UK. The current study aimed to understand the experiences of people with tics and their families in UK primary care and the referral process to secondary care for support, and how they perceive the support provided.

## Materials and methods

### Participants and recruitment

We hosted two cross-sectional surveys simultaneously via JISC Online Surveys, one of which was completed by adults with tics and the other by parents/carers on behalf of their child with tics. Participants were eligible for inclusion in the study if aged 18 years or over and if they had attended at least one appointment in the UK with a GP for their tics, or if they were a parent/carer of a child (aged 17 years or below) who had attended at least one appointment in the UK with a GP for their child’s tics. Participants could take part even if they or their child no longer experienced tics. Adults could participate regardless of whether support for tics was sought in childhood or adulthood. Participants were recruited through a study advert posted twice on the Instagram and Twitter pages of UK Tourettes Action (a national tic charity) and Neurodiverse (a neurodiversity consultancy service) and posted once on the Facebook page of Tourette Syndrome Support Group. Participants were recruited between 5th May 2022 and 14th July 2022. The study received ethical approval from the Division of Psychiatry and Applied Psychology Research Ethics Committee for the University of Nottingham, UK (Reference number: 2891).

### Study design and procedure

All questions were created de novo by the study team. There were six sections within the surveys incorporating a mix of multiple-choice questions, 4- and 5-point Likert scale ratings to indicate participants’ satisfaction or agreement with given statements (where 1= ‘Very satisfied’ and 5= ‘Very dissatisfied’, and 1= ‘Strongly agree’ and 4= ‘Strongly disagree’) and free-text responses. Response to all questions within a section was mandatory to move onto the next section, except for the free-text responses which were optional with no maximum or minimum character limit imposed. Incorporating both quantitative and qualitative elements in the surveys provided deeper insight into the thoughts and expectations participants had when accessing care for tics than would have been achieved using either methodology alone, which is a benefit of mixed-method surveys [37].

The topics covered included participant demographics, events occurring during the first GP appointment for tics, satisfaction with the GP’s knowledge and identification of tics, management offered by GPs, experiences of the referral process to secondary care, and overall satisfaction with the care provided by GPs. Skip logic was applied when the questions were not applicable to the participant. Both the adult and parent/carer surveys asked the same questions but were worded differently to be more appropriate for the individual completing the survey (i.e. “your tics” versus “your child’s tics”). A copy of the survey questions can be found in Additional File 1.

The surveys were critically reviewed by an expert working party which was created to aid the development of comprehensive national guidelines for the assessment and management of tics in England. The working party has 10 members including leading tic specialist psychologists, academic researchers, and patient and public involvement (PPI) members with lived experience of tics. Feedback was implemented in both surveys before they went live. Participants gave informed consent online before completing a survey response anonymously, with the option to enter a free prize draw after finishing the survey to win one of six Amazon.co.uk gift vouchers. Average time to completion across the two surveys was 21.1 min.

### Data analysis

With the prevalence of TS at 1% [2] and the general UK population consisting of approximately 68 million people, this provides a population size of 680,000 people with TS in the UK. Thus, powering the sample to achieve 95% confidence level and 5% margin of error, we calculated a sample size requirement of 384 across both survey groups.

Quantitative data from the surveys were analysed using IBM SPSS Statistics for Windows, Version 28.0. Analyses included descriptive statistics of adult and parent/carer responses both individually and combined, and Mann Whitney U tests comparing mean differences between adult and parent/carer responses as parametric assumptions for t-tests were not met. A significance level of 0.05 was used.

Themes from open-ended questions were developed at a semantic level using an inductive approach to thematic analysis. All analyses were conducted within the theoretical perspective of critical realism [38]. As little is known about what people experience when seeking support for tics from primary care, it was not possible to develop research-driven themes. The analysis was conducted by one researcher (CM) following the steps provided by Braun & Clarke [39]. When this study was conducted, CM was a UK fifth year medical student. CM gained experience in primary care during medical school, therefore was aware of the processes involved in clinical consultations and the referral process to secondary care. Her experience in this area facilitated the development of relevant survey questions despite being an outsider [40] with no personal experience of tics. Aware of the long-standing negative press targeting GPs which has been increasing in recent years [41–48], it was important to CM that the outcome of the project did not negatively impact public perception of the medical profession but still maintained an accurate representation of the participants who detailed their experiences to CM as a trusted researcher.

To verify the validity of the themes, KK and CLH independently conducted a thematic analysis on 10% of the dataset which was chosen using a random number generator. Discussions between all three researchers to negotiate the content and final themes and sub-themes ensured the qualitative findings were credible. Quotes from participants are denoted by speech marks and italics, which are followed by parentheses containing an ‘A’ (adult) or ‘PC’ (parent/carer) and a unique number.

Qualitative and quantitative data were analysed separately and then mixed during analysis in a methodological approach known as triangulation [49]. Both qualitative and quantitative data were given equal importance, as both sets of data were central to addressing the research questions.

## Results

We collected a total of 128 responses across both surveys. The adult survey received 34 responses whilst the parent/carer survey received 94 responses. One participant completed both surveys, so we merged these two responses and incorporated it into the parent/carer survey as the participant referred to their child with tics in their free-text responses. Thus, in total 127 responses were included in the quantitative analysis with 126 responses used in the qualitative analysis, as one participant did not provide free-text responses.

The majority of participants were white (93.7%) and over 70% lived in England (see Table 1). The gender split of the sample was equal, with roughly half identifying as a boy/man (48.0%) or girl/woman (48.9%).

**Table 1:** Demographic Information Collected from the Participants

Characteristic	Adult with tics (n = 33)	YP with tics (n = 94)	Combined (n = 127)
UK location, *n (%)*			
England	25 (75.8)	74 (78.7)	99 (78.0)
Wales	3 (9.1)	13 (13.8)	16 (12.6)
Scotland	5 (15.2)	5 (5.3)	10 (7.9)
Northern Ireland	0	2 (2.1)	2 (1.6)
Gender, *n (%)*			
Man/Boy	10 (30.3)	51 (54.3)	61 (48.0)
Woman/Girl	20 (60.6)	42 (44.7)	62 (48.9)
Non-binary	3 (9.1)	1 (1.1)	4 (3.1)
Ethnicity, *n (%)*			
White	33 (100)	86 (91.5)	119 (93.7)
Mixed or multiple ethnic groups	0	7 (7.4)	7 (5.5)
Asian or Asian British	0	1 (1.1)	1 (0.8)
Tic Diagnosis, *n (%)*			
TS	23 (69.7)	41 (43.6)	64 (50.4)
CTD	0	3 (3.2)	3 (2.4)
PTD	0	3 (3.2)	3 (2.4)
FND (functional tics)	1 (3.0)	1 (1.1)	2 (1.6)
Co-morbidity (may be more than one condition per participant), *n (%)*	31 (94.0)	58 (61.7)	89 (70.1)
Anxiety	24 (72.7)	31 (33.0)	55 (43.3)
OCD	14 (42.4)	22 (23.4)	36 (28.3)
Autism	8 (24.2)	20 (21.3)	28 (22.0)
ADHD	7 (21.2)	14 (14.9)	21 (16.5)
ID	1 (3.0)	8 (8.5)	9 (7.1)
Depression	17 (51.5)	2 (2.1)	19 (15.0)

YP = young people; TS = Tourette syndrome; CTD = chronic tic disorder; PTD = provisional tic disorder; FND = functional neurological disorder; OCD = obsessive compulsive disorder; ADHD = attention deficit/hyperactivity disorder; ID = intellectual disability

The mean age of first tic onset was 7.02 years (SD = 3.67) for YP and 9.72 years (SD = 5.54) for adult respondents (see Additional File 2). A total of 64 respondents (50.4%) had a diagnosis of TS, with very few diagnosed with other primary tic disorders or with functional tics (functional neurological disorder).

Within the sample, 70.1% reported at least one co-morbidity. The most common co-morbidity across both participant groups was anxiety, with a greater prevalence seen in adult respondents (72.7%) than young people (YP; 33.0%). Similarly, the rate of comorbid depression was much higher in the adult participants (51.5%) than in YP (2.1%).

### First GP consultation

The mean age at which YP had their first GP appointment for tics was 8.29 years (SD = 3.67 years), whilst for adult respondents this was 17.66 years (SD = 9.72 years) (see Additional File 2). Parents/carers were much less likely to delay presenting to primary care when their child had their first tic, with a mean delay of less than one year, compared to a 7.5-year delay calculated for the adult respondents. This difference was statistically significant, U = 794.00, p = < 0.001 (see Additional File 2).

**Fig. 1:**
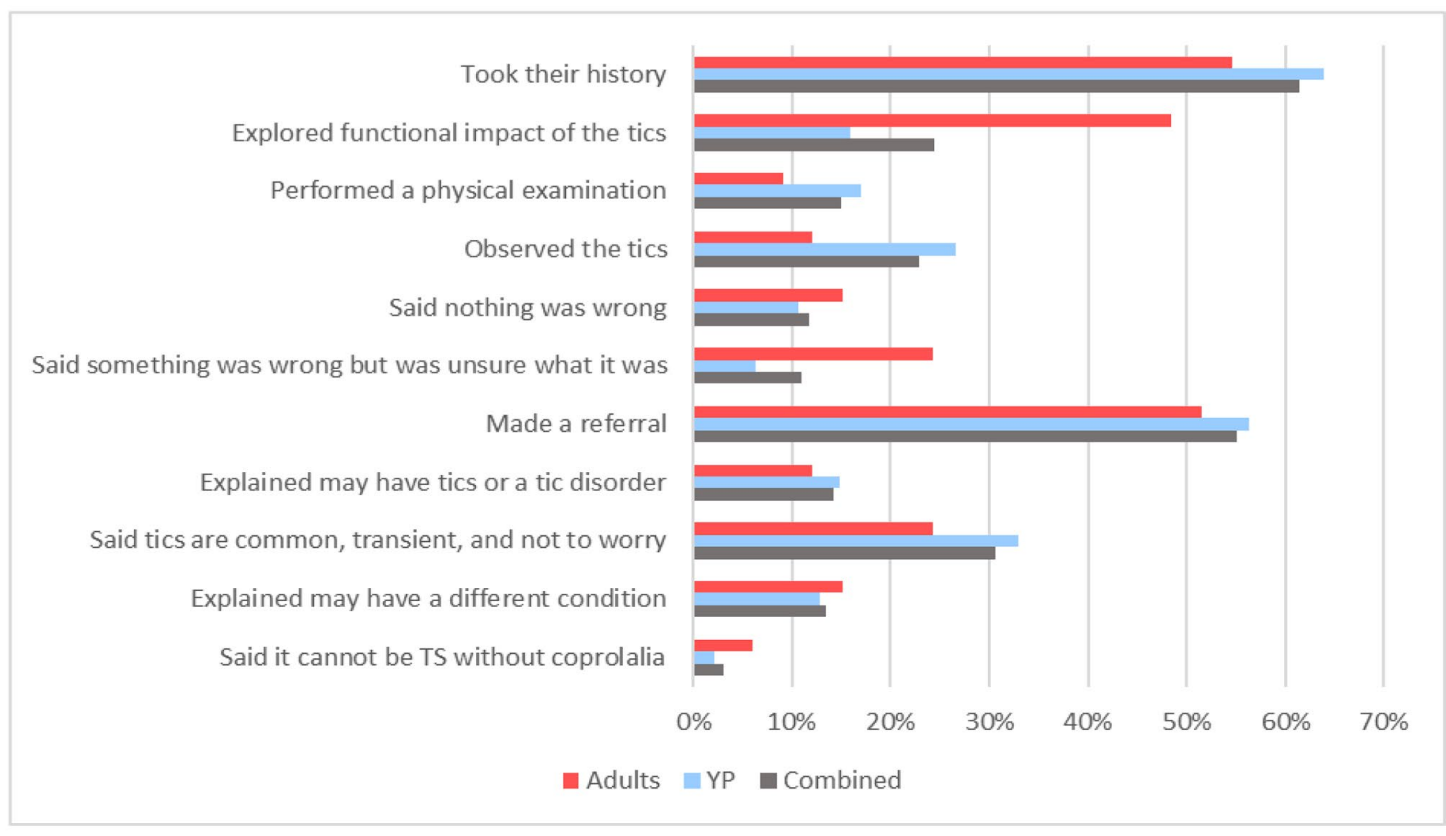
What the GP did During the First Appointment TS = Tourette syndrome; YP = young people

As shown in Fig. 1, the events most frequently reported to have happened during their first GP appointment were: the GP taking the patient’s history (61%), making a referral to secondary care (55%), and providing reassurance that tics are common and often transient and so should not be worried about (31%). The rates of these events being reported were consistent across both adults and parents/carers. However, GPs appeared more likely to explore the impact of the tics with the adult respondents (48%) compared to parents/carers (16%). Only 14% of participants reported that their GP mentioned tics or a tic disorder as a possible explanation for their experiences, with GPs more likely to be unsure of a diagnosis according to adult respondents (24%) compared to parents/carers (6%).

**Fig. 2:**
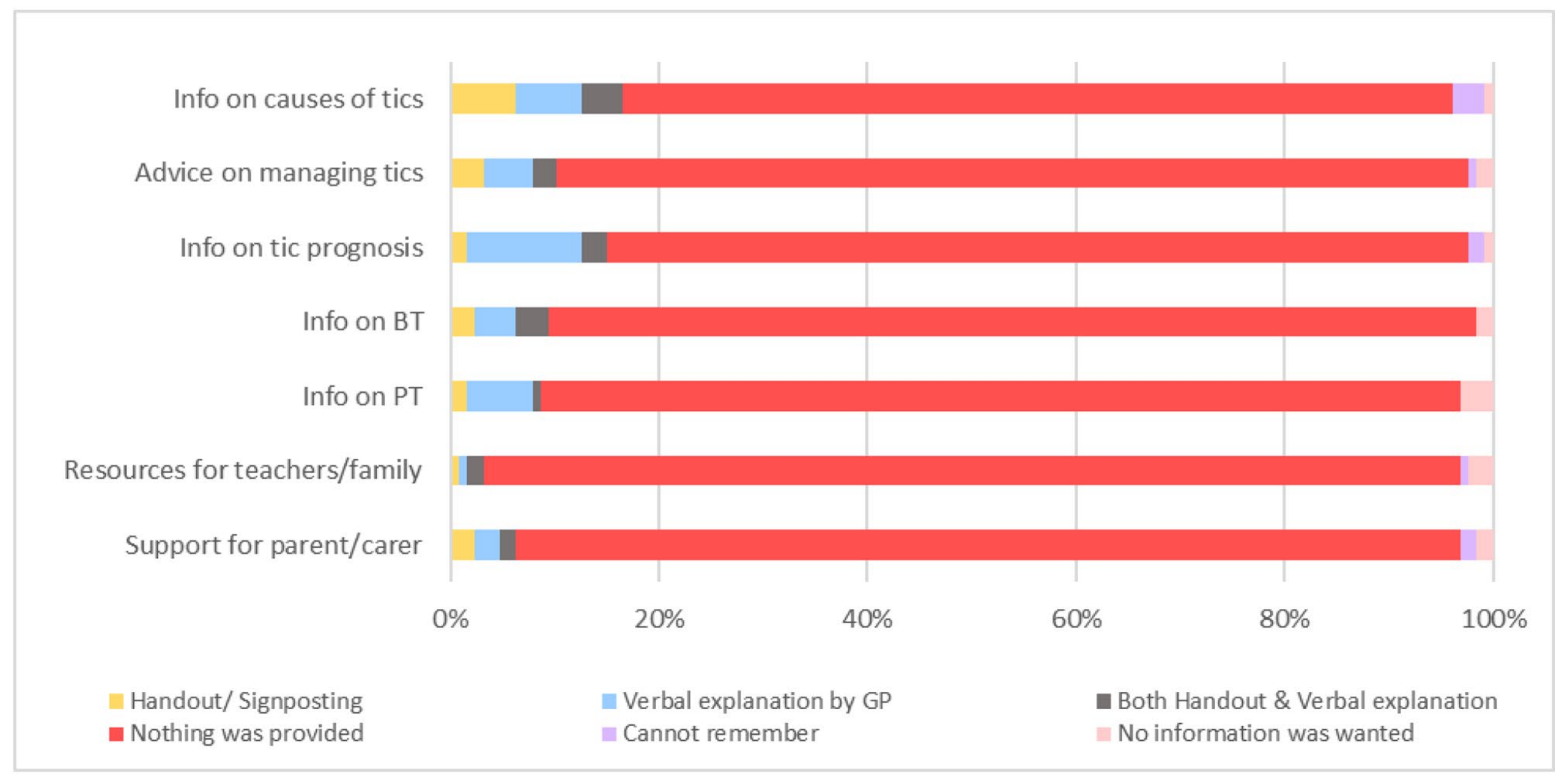
Immediate Management Provided in the First GP Appointment for Tics Info = information; BT = behaviour therapy; PT = pharmacotherapy

Figure 2 shows that psychoeducation (information) about tics and tic management were rarely provided by GPs in the first appointment. Over 80% of participants reported that they were not informed about the causes of tics or tic prognosis, with almost 90% not receiving information about treatment options or advice on how to better manage their tics. Many participants also reported that they were not advised on where to find resources for teachers and family members about tics (94%) or support for parents/carers (91%).

#### Getting a referral

In total, 78.7% (100/127) of participants had been referred to secondary care services at some point specifically for their tics. Adult respondents were also significantly older (M = 25.19 years, SD = 11.72 years) than YP (M = 9.42 years, SD = 3.42 years) when a referral was made to secondary care for tics, U = 149.50, p < .001.

### Perceptions of primary care for Tics

Participants held differing views on the satisfaction with the care they received for their tics from primary care. Whilst 21% were satisfied, 57% reported their dissatisfaction with the care provided by GPs (see Additional File 3). The qualitative data indicates that dissatisfaction arose due to a perception of GPs minimising tics and the struggles of patients and their families, whilst those who were satisfied felt supported because their GPs were compassionate and willing to collaborate with patients and their families to facilitate care. These two overarching themes of *Minimisation of Tics by GPs* and *Feeling Supported by GPs* are discussed in detail below (see Table 2 for a full list of the themes and sub-themes).

**Table 2:** Themes and Sub-themes

Themes	Sub-themes
*Minimisation of Tics by GPs*	Impact of Tics Not Taken Seriously
	Tics Misinterpreted as Other Conditions
	Having to Fight for a Referral
*Feeling Supported by GPs*	Pro-active and Compassionate GPs
	Easy Access to Referrals
	Working Collaboratively to Facilitate Care
*Issues with the Provision of Support by Secondary Care*	Poor Availability of Tic Services
	A Lack of Clear Referral Pathways for Tics

#### Minimisation of Tics by GPs

This theme describes how certain actions performed by their GP were perceived by participants as minimising their struggles and experiences. This included feeling misinformed about expected tic prognosis, poor appreciation by GPs regarding the severity and impact of the tics, GPs attributing tics to other conditions, and participants having to fight for a referral.

#### Impact of Tics not taken seriously

One recurring comment by participants concerned the advice given by their GP in the first appointment, in which they were told that the tics will go away eventually without intervention. Although tics may remit completely, this is not the case for all patients [9–11, 50, 51]. Thus, the advice given by their GP led to some feeling they had been given false hope:

“*I wish I wasn’t told they would probably go away, because I had been waiting for that to happen but instead they became more severe and more frequent*.” (PC, 93).

Additionally, many participants felt that their GP dismissed their tics as they were advised to carry on as normal despite the impact on quality of life. This approach continued past the first appointment with the GP, which led to participants later facing challenges in getting support even if the tics became more severe:

“*We felt that the GP had as little clue as to why my daughters previously quite innocuous tics could have turned so noticeable, life changing and violent overnight. Almost felt as if we weren’t believed.*” (PC, 72).

Sensing that the GP had “*little clue*” suggests a perception that the GP’s response was due to poor understanding of the changeable nature of tic severity and presentation. Families reported the negative impact this had on them:

“*…our daughter was acting like a chicken, shouting words out, kissing everything, swearing throwing herself on the floor. The list is endless, as a parent to see this happen to your child over night for no reason is very scary then to be pushed away by the doctors is even worse. Our lives changed forever that day.*” (PC, 42).

#### Tics misinterpreted as other conditions

This sub-theme discusses how some GPs misidentified tics as an aspect of other conditions. Most commonly this was anxiety, although epilepsy, behavioural dysregulation, ADHD, autism, allergies, and asthma were each also reported at least once.

This concept of poor identification of tics by GPs was reinforced by just over half of participants who perceived that GPs were unable to identify motor, phonic, simple, and complex tics well (see Additional File 4).

#### Having to fight for a referral

This sub-theme describes the difficulty some patients and parents/carers had when trying to get their GP to make a referral for the patient to secondary care. A few participants mentioned they had to push back against their GP’s advice and convince them that a referral was warranted:

“*…It was all down to me explaining multiple times my case and eventually getting referred to the neurologist who diagnosed me.*” (A, 25).

Having to push and explain themselves repeatedly highlights the struggle these participants faced to influence their GP’s decision and management plan. Adult respondents particularly appeared to have this struggle, with 20% more adult respondents than parents/carers reporting having ≥ 5 GP appointments before being referred to secondary care for their tics (see Additional File 5).

One reason for this difficulty in convincing their GP was revealed by an adult participant (A, 19), who had to attend multiple appointments arguing their case because their GP stated “*there was no [help] on offer*” locally for tics so a referral would be fruitless. Whilst this participant and others recognised that often “*there isn’t much help*” consistently available in secondary care for patients with tics, participants discovered through searching online that there were some professionals they could be referred to for support which the GP appeared unaware of. In these cases, patients had to present their findings to the GP to initiate the referral:

*“…I did a lot of research, gain the necessary information I needed to show drs that there was more they could do. After explain all the data I had collected dr agreed to take it to his next meeting to discuss my daughters case. Dr then contacted me to say that he would refer her to a neurologist…”* (PC, 20).

### Feeling supported by GPs

This theme highlights the positive experiences participants have had with their GPs. This includes feeling heard and helped by GPs, having a simple process for onwards referral, and GPs working alongside patients and their families to facilitate care.

#### Compassionate and pro-active GPs

Participants who spoke positively of their experiences in primary care stated they “*felt very much listened to*” (A, 29). GPs recognising distress, making appropriate referrals without hassle, and allocating sufficient time to explain what to expect moving forward were also appreciated by participants. Although a few participants noted that the support offered was limited in primary care as management frequently requires the input of a tic specialist, others spoke highly of GPs who were compassionate, chased referrals, and kept in regular contact:

“*The GP called regularly, chased referrals, wrote letters to support school… explained and discussed with me and my child and treated us like humans*” (PC, 69).

#### Easy access to referrals

This sub-theme captures the experiences of some participants who found it simple to get a referral to secondary care for their tics from their GP. Comments of this nature predominantly came from parents/carers, of which 54% reported being referred for their child’s tics after a single appointment (see Additional File 5). As described by participants, some GPs identified in the first appointment that they were unsure of the specific management needed by their patient and so advised that they should be seen by secondary care:

*“my [GP] was lovely. she had no idea what [my child was experiencing] but knew he needed to see a paed.”* (PC, 6).

#### Working collaboratively to facilitate care

Participants appreciated when their GP did not doubt their descriptions of the severity of the tics, recognising that participants “*knew [their] child better*” (PC, 88) and so were the better judge of the impact of tics on their child’s quality of life.

Additionally, some participants were actively involved in the referral process, aiding the GP by providing detailed information for the referral documentation. In circumstances where participants had researched which secondary care service they could be referred to, GPs were often reported to have acknowledged this effort and agreed to make referrals to these services. Whilst some noted their wish that the GP knew this information already, generally participants viewed this situation favourably:

*“…although I [felt the GP] could have done with more knowledge and resources, I was pleased that he respected my knowledge and agreed to make the referral for my son.”* (PC, 9).

### Experiences of secondary care for Tics

Of the 100 participants who have been referred, 89 had attended at least one appointment in secondary care before completing this survey. Most were referred to non-tic specialists, with only 27.9% seen by tic specialists (see Additional File 6). Adult respondents were much more likely (67%) to be seen by neurology services (both general neurologists and tic specialist neurologists) than YP for their tics. YP were slightly more likely to be seen by general paediatricians (33%) and general psychiatrists (30%), with a smaller proportion seen by tic specialist paediatricians, including paediatric neurologists (21%).

#### Issues with the provision of support by secondary care

The qualitative data highlighted certain issues with secondary care services for tics, which are described below.

#### Poor availability of Tic services

Despite their GP being willing to make a referral, some participants found they were unable to see a specialist due to a lack of specialists in their local area. Although this issue was experienced by both adult respondents and parents/carers, it appeared particularly problematic for the adult respondents who frequently reported having to travel up to 50 miles for their secondary care appointment (see Additional File 7). In addition, some adult participants were informed that their journey to diagnosis and support would be prolonged due to a dearth of adult services for tics:

“*I was told because of my age the NHS doesn’t really have any support network in place [for tics] so everything is going to be really slow in getting to see anyone or diagnosed. I don’t think this is fair at all just because of my age. I’m 30 years old*” (A, 2).

**Fig. 3:**
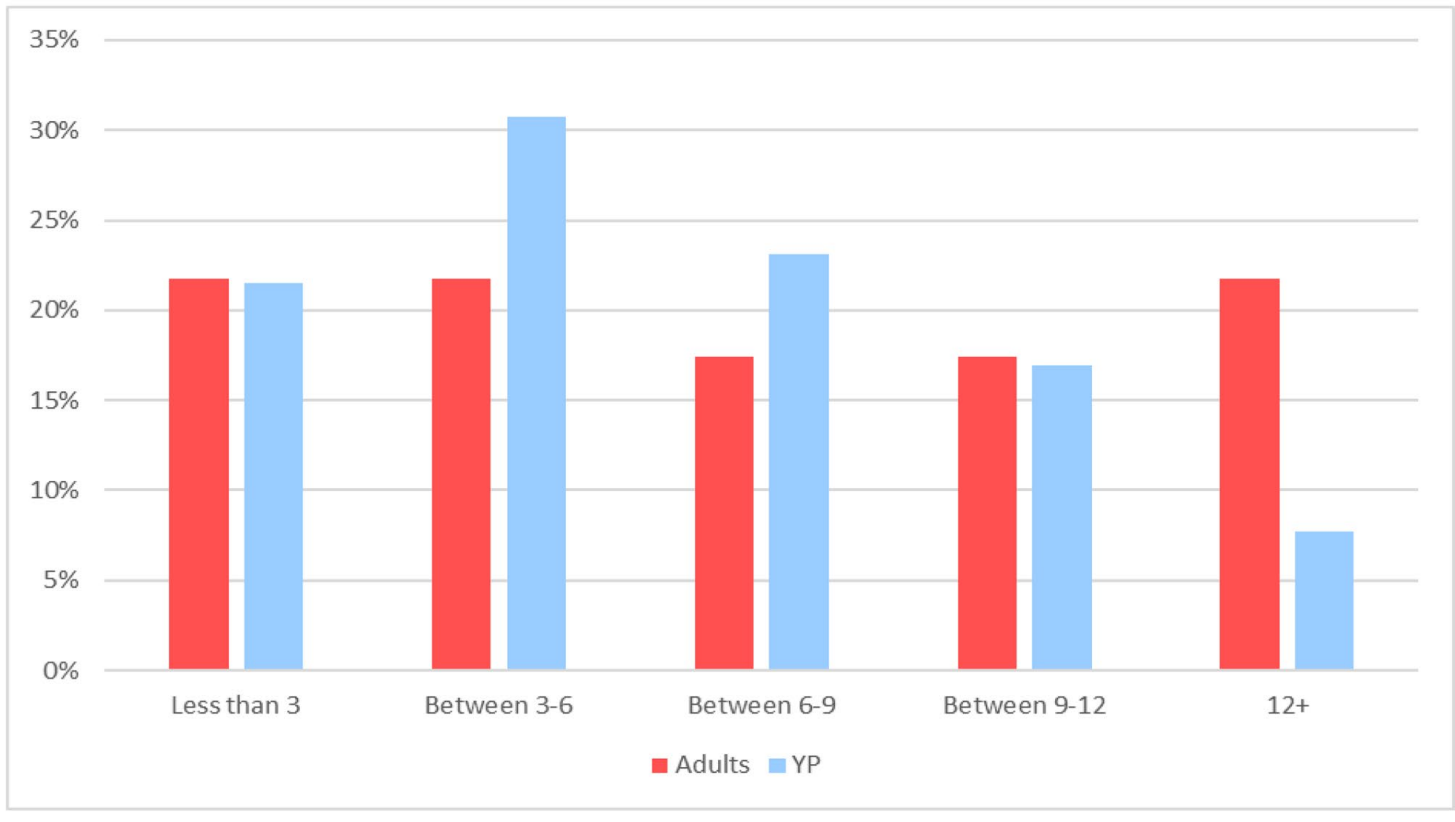
Number of Months Spent on the Waitlist for Their Secondary Care Appointment YP = young people

In those participants for whom a referral was made, respondents reported waiting many months for assessment in secondary care. Indeed, most YP were on the waitlist for 3–6 months whilst 22% of adult respondents reported waiting for longer than a year for an appointment (see Fig. 3), with one adult participant (A, 8) mentioning a 17-month wait for a neuropsychiatry assessment appointment. These long wait times meant in the interim patients and their families continued to struggle with no support, which often had negative consequences on their well-being:

“*…Unfortunately, we were then kind of left in limbo waiting for the pediatrician appointment with took 8 months… to be honest we felt very alone and scared. Our daughter went from a happy little girl to a very withdrawn sad girl.*” (PC, 81).

Almost one-third (29.1%) of participants reported accessing private healthcare to receive a diagnosis or management for tics. In some cases, participants paid for private care despite not having sufficient disposable income to afford it. Most stated that they turned to private care because they were not able to access support through the NHS in a timely manner. Others accessed care privately out of necessity as the NHS services they were referred to did not provide support for tics:

*“…the diagnosis we received was from us paying privately to see a pediatric neurologist. From our GP referal we are still awaiting an appointment from [CAMHS].”* (PC, 16).

#### A lack of clear referral pathways for Tics

Many participants found they were referred to several different services in secondary care, each stating they were unable to provide support:

“*…After being discharged [from CAMHS] at 16 there has been no help - I have been referred back and forth between neurology, neuropsychiatry, neuropsychology and psychology, all saying there are no specialists, and they can[‘t] help me.*” (A, 26).

“*My child was referred only to children’s paediatrician who could not advise. They referred to CAMHS who couldn’t help…*” (PC, 91).

In total, 21% (19/89) of participants mentioned receiving no support for tics from the secondary care services they had been referred to. In some cases, parents/carers returned to their GP who suggested they contact their child’s school for help, who responded that they could not provide support without specialist input. As a result, participants felt they were caught in a never-ending loop between the school and NHS services, with both “*taking no accountability*” (PC, 4) for providing support for tics.

## Discussion

This study provides insight into the experiences of adults and parents/carers of YP in both primary care and the referral process to secondary care for tics. The results revealed mixed, albeit mostly negative, experiences in primary care. Whilst some participants felt supported by GPs who were understanding, proactive, and provided care through working with patients and their families, many felt their care was hindered by their GP’s poor appreciation for the impact of tics and by the initial difficulty they experienced in getting a secondary care referral. Although 78.7% were referred for their tics, a lack of specific tic referral pathways meant there was a poor understanding among patients, GPs, and secondary care professionals regarding which specialty caters to tics. Many participants were referred back and forth between secondary care services, often without any support provided. Long waitlist times and a dearth of NHS tic specialists available locally compounded this issue, with almost one-third resorting to accessing care privately. The surveys did not explore perceptions or experiences with tertiary care.

Regarding the first GP consultation for tics, the events occurring during the appointment were universal among both adult respondents and YP, which included history-taking, referrals to secondary care, and reassurance that tics are common and likely transient. However, exploration of the impact on daily functioning of tics was reported less frequently by parents/carers than adult respondents. The reason for this is unclear, but it could be due to misconceptions about transient tics. On average, YP presented to primary care within a year of first tic onset whilst adult respondents waited 7.5 years before making a GP appointment for their tics, so tics were potentially identified as transient and likely to remit soon in YP and persistent in the adult respondents. As transient tics are often mild [2, 9], this may have resulted in GPs not fully considering the impact on YP and their parents/carers who subsequently felt dismissed. As current guidance recommends referrals based on tic severity and impact rather than persistence [21, 23], this reinforces the importance of GPs asking about the impact on daily functioning even for transient tics.

Additionally, in line with previous reports of minimal information giving by HCPs [29], very few participants received information about tics or tic management options from their GP. Understanding information pertinent to their medical condition is essential for good health literacy in patients [52, 53] and is associated with improvements in health-related quality of life, depression, and anxiety [54]. Psychiatric conditions are commonly co-morbid with TS, with up to one-third also diagnosed with mood or anxiety disorders [13, 16, 17]. This is important as the present study found that the prevalence of mental health co-morbidities in the adult respondents was high, suggesting psychoeducation as an early intervention could offer a potential avenue to reduce the risk of these conditions developing. Thus, this appears to be an area where tic management in primary care could be improved.

Regarding information provided by GPs on tic prognosis, some participants stated that such discussions were falsely reassuring. GPs were often reported to advise that tics in children would likely remit eventually, in line with previous expert opinion on tic prognosis [6–8]. However, more recent evidence challenges this, suggesting that tics are more likely to persist than completely remit but often become less severe with less impact on quality of life with time [9, 11, 50, 51]. Indeed, participants who were desperate for the tics to disappear later felt hopeless when this did not occur. This emphasises the importance of clearly discussing the possibility of tics persisting in all patients even if remittance does eventually occur, alongside providing advice on who to contact should the tics worsen.

Previous research discovered a perception among patients that HCPs lack knowledge about tics [29, 30]. The present study found that the perceived gaps in GPs’ knowledge included identifying different types of tics, distinguishing tics from other conditions such as anxiety, and knowing where to send referrals. Existing literature has demonstrated that limited knowledge about tics in professionals seeing patients can delay accurate diagnosis and therefore treatment [55, 56]. Indeed, in the present study, some participants reported researching which specialty they could be referred to and presenting their findings to their GP before a referral was made. This suggests a lack of clear and well-known referral pathways for tics from primary care, which could explain why accessing specialist management for tics has previously been reported as difficult [29]. Many secondary care clinicians spanning CAMHS, neurology, and paediatrics appear to support this idea, as at their referral appointment patients were informed either that they were not referred to the correct service or that the service they were referred to does not provide support for tics. Research exploring the current referral pathways available for tics from the perspectives of both primary and secondary care professionals, including its challenges and recommendations for improvement, would be beneficial to confirm this as a cause of difficulty in accessing support.

Although most patients were referred to secondary care for their tics, only 27.9% of participants were referred to tic specialists. This is more than double the referral rate to tic specialists reported previously [57], though this difference could be explained by the previous study having a small sample size involving only one NHS trust. Adult respondents most commonly reported being assessed by general neurologists, whilst YP tended to attend appointments with CAMHS or general paediatrics. While mode waitlist time for YP was 3–6 months, in line with previously reported average UK waitlist times [31], adult respondents appeared to have waited longer, with 22% waiting over a year for a secondary care appointment. National targets require patients to have consultant-led treatment initiated within 18 weeks from receipt of the referral [58], which many of the referrals for adult respondents appeared to be exceeding. Indeed, neurology outpatient waitlist times have been increasing over the last decade, with 23% of patients in England waiting over 18 weeks to receive management by a neurologist in March 2019 [59]. This can be partially explained by the severe shortage of full-time equivalent consultant neurologists in the UK where there is 1 per 91,175 of the population, almost four times fewer neurologists per capita than in comparable European nations [60]. This, in addition to increasing demand for neurological services [58], has resulted in poor access. Unfortunately, staffing crises are present in many specialties in the UK, including GP [61], paediatrics [62, 63], and psychiatry [64], leading to difficulties delivering high quality care in a timely manner for both adults and YP with tics who consequently struggle without support. Discussions regarding how to reduce waitlist times for patients with tics should be prioritised, including evaluating the feasibility of funding fully staffed tic specialist services similar to those created for ADHD [65]. A summary of our key findings and recommendations for primary care clinicians and secondary care services can be found in Table 3.

**Table 3:** A Summary of our Findings and Recommendations

**Key points and recommendations for primary care clinicians:**
- Tics are common in school children and can persist into adulthood - The impact of tics should be explored in all patients, even if tics are new-onset, as this informs the need for a secondary care referral - Information about tics should be provided to patients (and parents/carers if appropriate), to include general tic prognosis and a brief overview of potential treatment options - Tics are unlikely to completely remit in most people but typically prognosis is good. With time, tics often become less severe and have a smaller impact on quality of life and a minority experience worsening of their tics; manage patient and parental expectations accordingly
**Key points and recommendations for secondary care services**:
- Patients and family members raised the following concerns of secondary care: o Long waitlist times, particularly for adults o Being referred to multiple secondary care services (neurology, paediatrics, psychiatry) sequentially as each said they do not offer support for people with tics o Being discharged from secondary care after diagnosis with no support provided - There appears to be a paucity of NHS clinicians with a specific interest in tic management across the UK - Greater clarity is needed on which secondary care services can receive referrals for tics and manage these patients appropriately – consider formation of dedicated NHS tic services

### Strengths and limitations

Although we did not meet our target sample size, we analysed the free text responses using thematic analysis and reached saturation. Combining quantitative and qualitative items enabled a more comprehensive picture to be gained with regards to patients experiences of accessing NHS services for support for tics. Despite the anonymity provided by online surveys enabling participants to describe both positive and negative experiences which they otherwise may have been uncomfortable detailing if interviews or focus groups were used, it is likely that recall and self-selection bias are a limitation of the present study. Furthermore, as members of TA are more likely to have long-standing tics, the experiences of individuals navigating primary care with transient tics are possibly underrepresented in this study. Recruiting directly from GP practices via database searches of patient records for comments on tics, or auditing services for numbers of consultations involving tics, could usefully supplement the findings of this project.

Despite the strengths of this study, there were several limitations. As this project was conducted as part of a master’s degree, there was limited ability to involve multiple perspectives (e.g. psychologists) in the analysis of the data due to time constraints. Similarly, we achieved a smaller sample size than originally planned as a result of the limited timeframe within which the project could be completed in order to meet the deadlines instituted by the degree course. This unfortunately meant that we were limited in our ability to extend the duration of the surveys being live and to gain approval from the ethics committee to advertise the project through additional means to recruit more participants. Half of all participants were women/girls which contrasts prevalence ratios reported for tics in men and women, although analyses assessing whether there were gender differences in the findings were not statistically significant. Additionally, some participants mentioned specific experiences in secondary care unprompted such as receiving no support for tics from the secondary care services they had been referred to. We have included this information in the study where relevant, but the numbers generated may not reflect the true prevalence of this experience as it was not explicitly asked for in the surveys. We also cannot be sure if the adult respondents were reflecting on their childhood experiences or experience as an adult as this was not assessed in the survey. Nevertheless, given the mean age of first consultation for tics with a GP and mean age at referral for the adult respondents, it is likely the results from adult respondents reflect the experiences of adults with tics.

Whilst outside the remit of this study, further research could consider going into more depth into the experiences of tic treatment itself (i.e. in secondary care services and/or private practices). Finally, due to the potentially hereditary nature of tics, further research could also explore whether there are any differences experienced in accessing support if there is a family history of tics versus no family history.

## Conclusion

Adults and parents/carers of YP with tics described generally negative experiences of seeking support for tics in the UK in this study. Many felt their concerns were downplayed by their GP and received no information about tics or tic management, with some struggling to obtain a secondary care referral for further support. Although most were referred to secondary care, referrals were predominantly to non-tic specialists. A lack of clear tic referral pathways from primary care, long waitlist times, and a lack of support offered at primary and secondary care referral appointments meant accessing management for tics from knowledgeable specialists was delayed for many individuals, with one-third resorting to private care for their tics. As little research has previously focused on the patient experiences, accessibility, and availability of support for tics from primary care, this study highlights some key areas where improvements to NHS services for tics can be made for the benefit of this patient population.

## Electronic supplementary material

Below is the link to the electronic supplementary material.

Supplementary Material 1: Survey for adult and parent/carer participants.

Supplementary Material 2: Table comparing the key stages of the healthcare journey of the young people and adult participants.

Supplementary Material 3: Figure of participants’ satisfaction with various aspects of the care received from GPs for tics.

Supplementary Material 4: Figure showing the perception of participants in how able GPs were in identifying tics.

Supplementary Material 5: Figure showing the number of GP appointments attended before a secondary care Referral was made.

Supplementary Material 6: Figure showing secondary Care specialists which the participants have been referred to.

Supplementary Material 7: Figure showing the distance travelled (in miles) for the specialist appointment for tics.

## Data Availability

The datasets generated during the current study are available from the corresponding author upon request.

## Abbreviations

ADHD: Attention/deficit hyperactivity disorder
CAMHS: Child and Adolescent Mental Health Service
GP: General Practitioner
HCP: Healthcare Professional
NHS: National Health Service
NICE: National Institute for Health and Care Excellence
OCD: Obsessive compulsive disorder
PC: Parent/carer
PPI: Public and Patient Involvement
TS: Tourette Syndrome
YP: Young people
