# Supplementary material for: Patients’ experience of accessing support for tics from primary care in the UK: an online mixed-methods survey
Source: BMC Health Serv Res. 2023 Jul 24;23:788. doi: 10.1186/s12913-023-09753-5 (PMC10367334; doi:10.1186/s12913-023-09753-5)
Supplement: Supplementary file 1 — Supplementary Material 1: Survey for adult and parent/carer participants. [file 12913_2023_9753_MOESM1_ESM.docx]

# Additional File 1

#
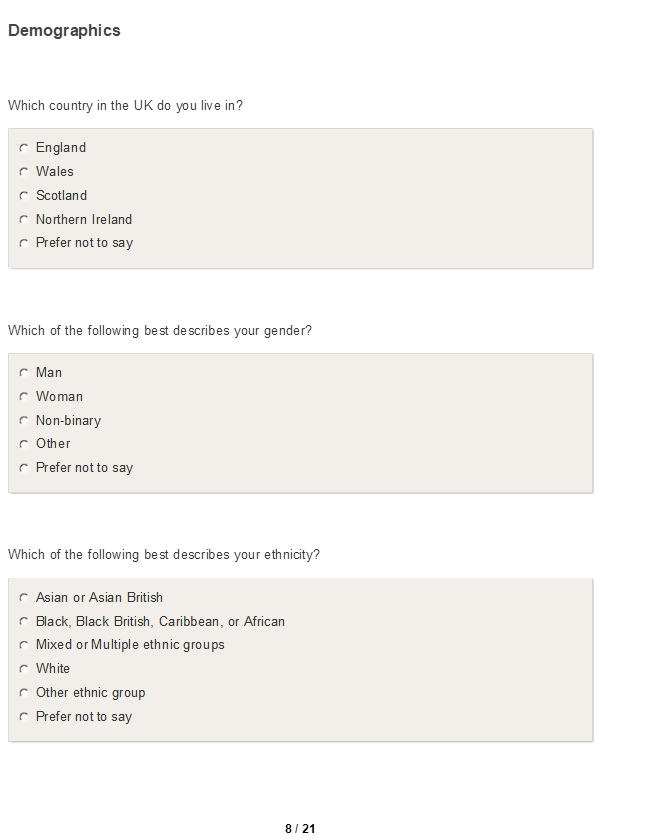
Adult Survey

#


*The More info section above contains the following message:*

*“For reference:    Motor tics are tics that involve movement of any body part.*

*Phonic tics are tics that cause sound (words, phrases, or any other noise).*

*Simple tics are tics that involve one muscle group such as sneezing, coughing, eye twitching, or tics that cause noises (not words or phrases).*

*Complex tics can be multiple simple tics occurring at the same time. They can also be tics involving multiple muscle groups such as suddenly bending over, or tics where you say words or phrases spontaneously.”*

*The More info section above contains the following message:*

“NOTE: ‘Privately’ means that you had to pay directly for the appointment(s) with the tic specialist.”

**Parent/Carer Survey**

*The More info section above contains the following message:*

*“For reference:    Motor tics are tics that involve movement of any body part.*

*Phonic tics are tics that cause sound (words, phrases, or any other noise).*

*Simple tics are tics that involve one muscle group such as sneezing, coughing, eye twitching, or tics that cause noises (not words or phrases).*

*Complex tics can be multiple simple tics occurring at the same time. They can also be tics involving multiple muscle groups such as suddenly bending over, or tics where you say words or phrases spontaneously.”*

*The More info section above contains the following message:*

“NOTE: ‘Privately’ means that you had to pay directly for the appointment(s) with the tic specialist.”
