# Supplementary material for: Patients’ experience of accessing support for tics from primary care in the UK: an online mixed-methods survey
Source: BMC Health Serv Res. 2023 Jul 24;23:788. doi: 10.1186/s12913-023-09753-5 (PMC10367334; doi:10.1186/s12913-023-09753-5)
Supplement: Supplementary file 2 — Supplementary Material 2: Table comparing the key stages of the healthcare journey of the young people and adult participants. [file 12913_2023_9753_MOESM2_ESM.docx]

# Additional File 2

| Comparing the key stages of the healthcare journey of the young people and adult participants. | | | | | | | |
| --- | --- | --- | --- | --- | --- | --- | --- |
|  | | N | Mean (SD) | Mean rank | U | Z | *p* value |
| Age at First Tic Onset in | Adults | 32 | 9.72 (5.54) | 78.34 | 1029.00 | -2.674 | .007 |
|  | YP | 94 | 7.02 (3.67) | 58.45 |  |  |  |
| Age at First GP Appointment for Tics in | Adults | 32 | 17.66 (8.57) | 95.94 | 466.00 | -5.832 | <.001 |
|  | YP | 94 | 8.29 (3.63) | 52.46 |  |  |  |
| Age at Referral to Secondary Care for Tics in | Adults | 27 | 25.19 (11.72) | 81.46 | 149.50 | -6.503 | <.001 |
|  | YP | 73 | 9.42 (3.42) | 39.05 |  |  |  |
| Delay Between Age at First Tic Onset and Age at First GP Appointment for Tics in | Adults | 31 | 7.48 (9.45) | 80.37 | 794.00 | -3.775 | <.001 |
|  | YP | 90 | 0.92 (1.18) | 52.33 |  |  |  |
| Delay Between Age at First Tic Onset and Age at Referral to Secondary Care for Tics in | Adults  YP | 25  70 | 14.44 (12.40)  2.31 (2.16) | 68.66  40.62 | 358.50 | -4.411 | <.001 |
| Delay between Age at First GP Appointment for Tics and Age at Referral to Secondary Care for Tics in | Adults | 25 | 4.88 (7.50) | 53.24 | 744.00 | -1.203 | .229 |
|  | YP | 70 | 1.04 (1.48) | 46.13 |  |  |  |
| YP=young people. | | | | | | | |
