# Supplementary material for: Patients’ experience of accessing support for tics from primary care in the UK: an online mixed-methods survey
Source: BMC Health Serv Res. 2023 Jul 24;23:788. doi: 10.1186/s12913-023-09753-5 (PMC10367334; doi:10.1186/s12913-023-09753-5)

# Additional File 3

Participants’ Satisfaction With Various Aspects of the Care Received From GPs for Tics.


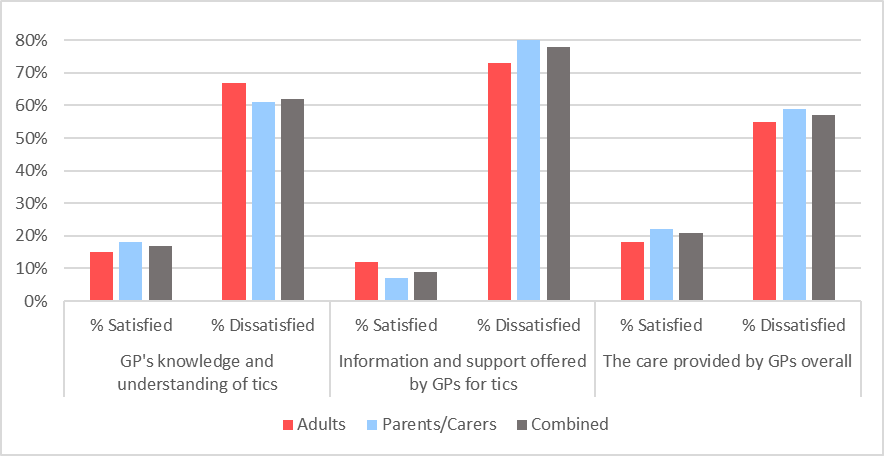

Supplement: Supplementary file 3 — Supplementary Material 3: Figure of participants’ satisfaction with various aspects of the care received from GPs for tics. [file 12913_2023_9753_MOESM3_ESM.docx]
