# Supplementary material for: Patients’ experience of accessing support for tics from primary care in the UK: an online mixed-methods survey
Source: BMC Health Serv Res. 2023 Jul 24;23:788. doi: 10.1186/s12913-023-09753-5 (PMC10367334; doi:10.1186/s12913-023-09753-5)
Supplement: Supplementary file 4 — Supplementary Material 4: Figure showing the perception of participants in how able GPs were in identifying tics. [file 12913_2023_9753_MOESM4_ESM.docx]

# Additional File 4


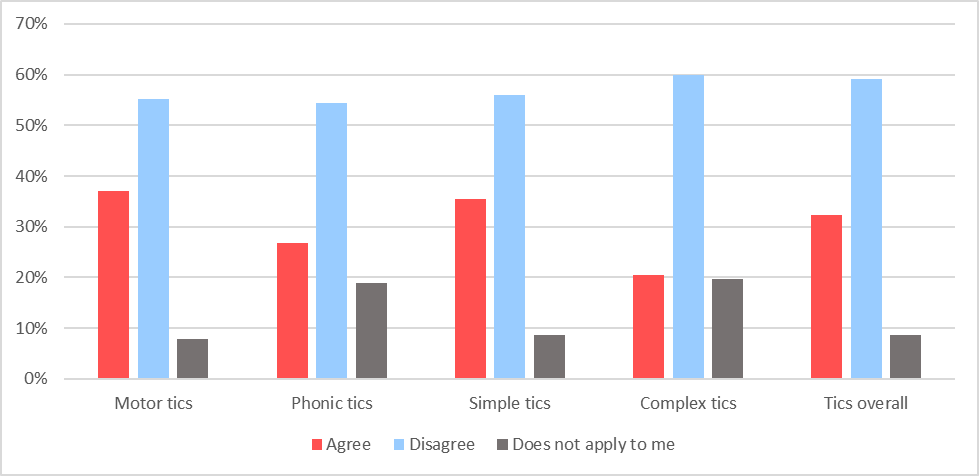
 The Perception of Participants in how Able GPs Were in Identifying Tics.

Participants rated how strongly they agreed with statements regarding how well their GP was able to identify each type of tic and tics overall.
