# Supplementary material for: Patients’ experience of accessing support for tics from primary care in the UK: an online mixed-methods survey
Source: BMC Health Serv Res. 2023 Jul 24;23:788. doi: 10.1186/s12913-023-09753-5 (PMC10367334; doi:10.1186/s12913-023-09753-5)
Supplement: Supplementary file 5 — Supplementary Material 5: Figure showing the number of GP appointments attended before a secondary care Referral was made. [file 12913_2023_9753_MOESM5_ESM.docx]

# Additional File 5


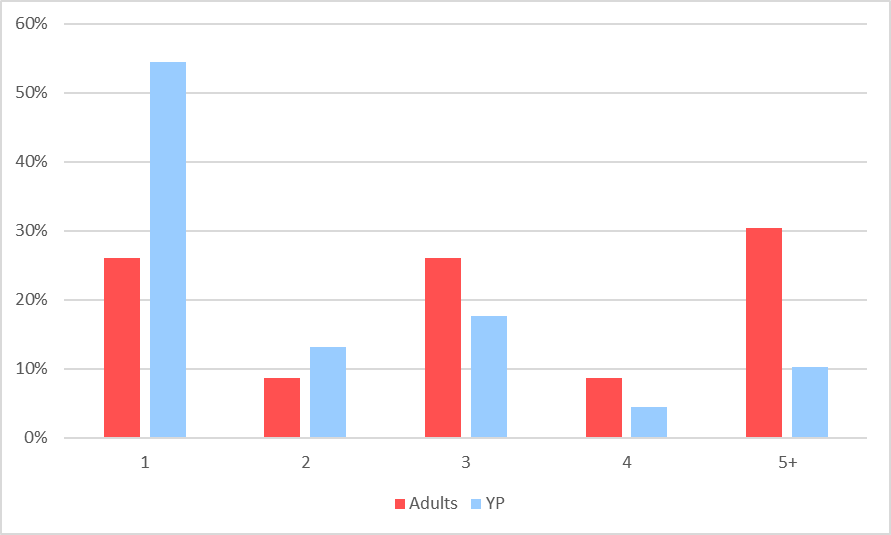
Number of GP Appointments Attended Before a Secondary Care Referral was Made.

YP=young people.
