# Supplementary material for: Patients’ experience of accessing support for tics from primary care in the UK: an online mixed-methods survey
Source: BMC Health Serv Res. 2023 Jul 24;23:788. doi: 10.1186/s12913-023-09753-5 (PMC10367334; doi:10.1186/s12913-023-09753-5)
Supplement: Supplementary file 6 — Supplementary Material 6: Figure showing secondary Care specialists which the participants have been referred to. [file 12913_2023_9753_MOESM6_ESM.docx]

# Additional File 6


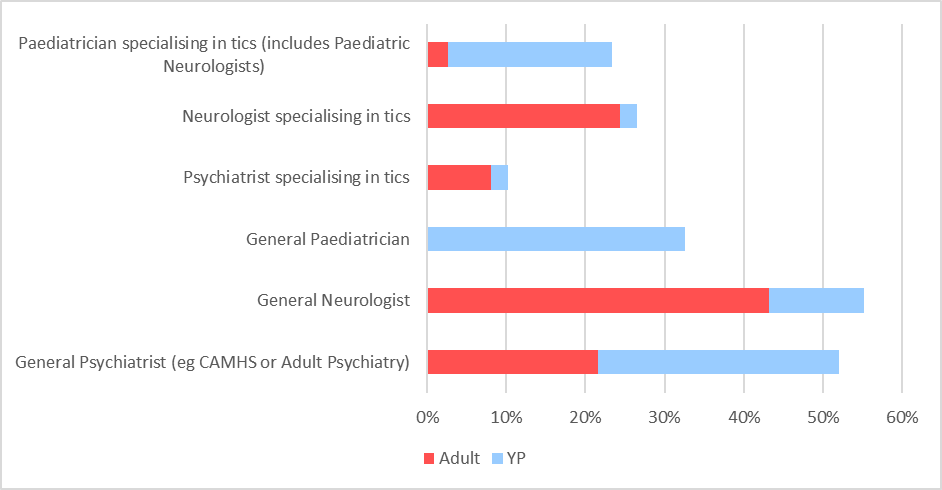
Secondary Care Specialists Which the Participants Have Been Referred to.

Participants were able to select multiple options if they had been referred to more than one. YP=young people.
