# Supplementary material for: Patients’ experience of accessing support for tics from primary care in the UK: an online mixed-methods survey
Source: BMC Health Serv Res. 2023 Jul 24;23:788. doi: 10.1186/s12913-023-09753-5 (PMC10367334; doi:10.1186/s12913-023-09753-5)
Supplement: Supplementary file 7 — Supplementary Material 7: Figure showing the distance travelled (in miles) for the specialist appointment for tics. [file 12913_2023_9753_MOESM7_ESM.docx]

# **Additional File** 7


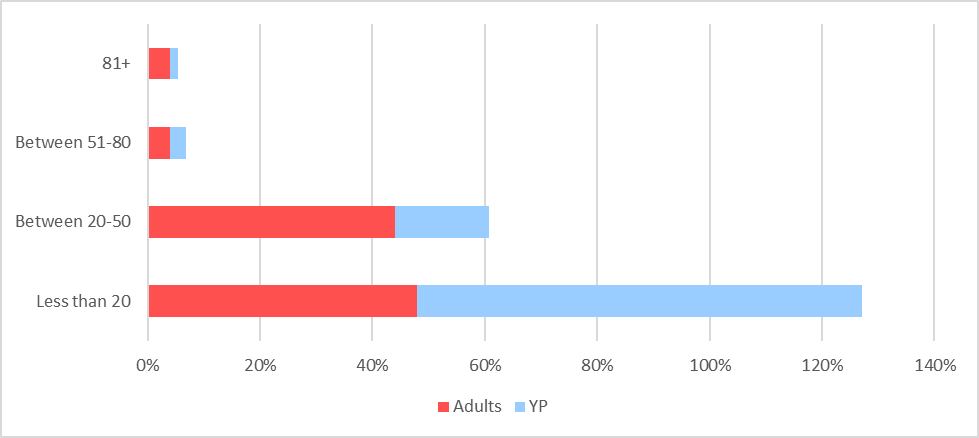
Distance Travelled (in Miles) for the Specialist Appointment for Tics.

Participants who had not had the appointment yet answered based on how far they will have to travel. YP=young people.
